# Rock physics-based approach to assess the effectiveness of cement-based backfill materials in coal mining

**DOI:** 10.1371/journal.pone.0333364

**Published:** 2025-10-08

**Authors:** Ma Liqiang, Qazi Adnan Ahmad, Md Mojahidul Islam, Nasir Khan, Hassan Nasir Mangi, Naser Golsanami

**Affiliations:** 1 School of Mines, China University of Mining and Technology, Xuzhou, China; 2 Key Laboratory of Xinjiang Coal Resources Green Mining, Ministry of Education, Xinjiang Institute of Engineering, Urumqi, China; 3 Key Laboratory of Deep Coal Resource Mining, China University of Mining & Technology, Xuzhou, China; 4 College of Energy and Mining Engineering, Shandong University of Science and Technology, Qingdao, China; 5 Department of Geology, Bacha Khan University Charsadda, KPK, Pakistan; 6 Department of Mechanical Engineering (Well Engineering), International College of Engineering and Management, Muscat, Oman; China University of Mining and Technology, CHINA

## Abstract

Coal production for energy generation results in series of ecological and environmental degradation in mining areas. backfilling of goafs is considered as a suitable method for the mitigation of aforementioned problems. Being economical, sustainable and safe to use for filing voids, supporting ground and managing the waste, cemented paste backfill (CPB) is the best choice for underground mining. However, the prediction of mechanical and elastic properties of CPB based on varying composition and curing time always remain a complex and difficult job. Traditional empirical studies although advantageous are tedious, lengthy and incapable to incorporate important aspects of geo-mechanical instabilities of backfill materials. In current study, a numerical approach is applied to simulate and evaluate the compressional (Vp) and shear (Vs) wave velocities, along with Vp/Vs ratio of CPB materials with varying flyash and biochar concentration at different curing intervals (3, 7, 14, 21 and 28 days). By applying a rock physics model, the proposed methodology offers a unique way to examine the effect of curing time and compositional variation on the elastic wave properties of CPB, ultimately offer insights into the internal structure and strength development over time. Simulation outcomes show that a high concentration of flyash and low concentration of biochar exhibit best elastic responses, with high Vp, Vs and low Vp/Vs ratio serving as precise indicator of materials mechanical integrity and heterogeneity. Eventually this study provides a time-efficient, cost-effective, and non-destructive method to analyze multiple backfill compositions in situations where laboratory work is lacking. Furthermore, this study promotes the use of rock-physics modeling at preliminary stage of material evaluation, with emphasizing its utility, productivity, and capability to enhance prediction precision in geo-mechanical investigations.

## Introduction

To meet the energy requirements, extensive coal mining raises various concerns regarding safety, environmental sustainability and resource efficiency in underground mining [1,2].

For the mitigation of these problems, backfilling of goafs with suitable material enhance the safety of the area by providing environmental sustainability and ensuring the efficiency of underground resources [3,4]. Optimizing backfill material to provide structural reinforcement to overlying strata, reduce subsidence and maintain the integrity of infrastructure located above mined areas is the key aspect of backfilling [5,6].

Due to their extensive availability, cost-effectiveness, environmental sustainability, suitable mechanical strength, enhanced mine safety and regulatory support, material such as coal gangue, fly ash and other mining waste are considered most suitable for backfilling [7,8]. However, it is necessary to evaluate these materials in term of their stiffness, rheological behavior and mechanical strength, so that the safety and structural stability of various mining and civil engineering applications can achieved [9,10].

In recent years, a number of empirical studies have been conducted to evaluate the mechanical strength of the backfill material. Liu et al. emphasized that during fully mechanized coal mining operations the mechanical behavior of material is essential to control the ground movement [11]. Similarly, Yufan et al. analyzed the creep hardening-damage characteristic through uniaxial compressive strength (UCS) to evaluate the mechanical strength of backfill material [12,13]. Significance of UCS testing is highlighted in an experimental study by Huang et al. for strength evaluation of backfill material under varying condition [14,15]. The outcomes reveal that backfill material having low strength can results into both surface and subsurface structural instability.

The strength of the backfill material can be enhanced through proper curing technique and applied stress during curing, which is essential for the durability of backfill materials under varying subsurface mining conditions [16]. Material composition is another factor that influences the mechanical and structural integrity of backfill materials [17]. A comprehensive analysis of backfill material with varying compositions, particularly their strength, is essential for ensuring the durability of mining and civil engineering projects [18].

UCS testing remains a fundamental method for assessing the mechanical reliability of backfill materials under varying subsurface conditions [19]. Although the conventional UCS method is efficient in estimating the mechanical strength of the backfill materials, it is a labor-intensive and time taking process [20]. This makes it less effective for rapidly evaluating mechanical strength of material having highly variable composition in a large-scale or real time applications [21,22]. In order to overcome such difficulties, development of an efficient and more predictive approach is essential. A rock-physics based numerical integration of mechanical strength of material with the physical properties (i.e., mineralogy, porosity, density and elastic moduli) could be a more robust and powerful alternative. Unlike purely empirical, experimental or soft computing methods, rock-physics based methodology provide a better understanding of how microstructural properties and material composition influence the mechanical response of backfill materials.

In order to establish a correlation between the mechanical properties (seismic velocities) and the inherent characteristics of backfill materials, such as porosity, permeability, elastic moduli, mineral composition and curing time, a systematic analysis is required. This study employs rock physics model and performs seismic analysis, by evaluating the effect of variations in material composition such as decreasing flyash concentration and increasing biochar concentration (Table 1) on the elastic and mechanical behavior of cement-based backfill materials. This rock physics-based approach offers a reliable methodology to predict material’s stiffness, strength and potential failure mechanisms by simulating the variations in compressional wave velocity (Vp) shear wave velocity (Vs) and Vp/Vs ratio [23,24]. Although this approach will enable the development of a real-time, non-destructive evaluating technique with the ability to promote safe and more efficient practices both in mining and civil engineering applications. However, the conclusion of current investigation may be site dependent which may not be smoothly applicable to different geological conditions in the absence of further refinements.

**Table 1:** Composition of Backfilling Samples.

QR Group	Fly ash (g)	Coal gangue (g)	Biochar (g)	Cement (g)	Plasticizer (g)	Water (g)	Total (g)
CMG-1	114	150	0	30	6	87	387
CMG-2	108	150	6	30	6	87	387
CMG-3	102	150	12	30	6	87	387
CMG-4	96	150	18	30	6	87	387
CMG-5	90	150	24	30	6	87	387

## Methodology

The scrutiny and evaluation of materials used for backfill in mining operations, such as fly ash, coal gangue, biochar, and cement in a subsurface environment are critical to ensure stability and avoiding the risk of subsidence [25]. The current research takes advantage of an improved poroelastic theory developed by Biot, derived from geophysical studies of subsurface porous media and specifically adapted to the examination of heterogeneous backfill materials [26,27]. This work employs a refined poroelastic theory developed by Biot, which was generated from geophysical investigations of subsurface porous media and applied to the analysis of several different backfill materials. Our approach employs a numerical model that represents a porous medium saturated with water. The physical interactions between these solid particles and pore fluids are modeled using the framework provided by Biot’s poroelastic theory, which allows an accurate simulation of how compressional (V_P_) and shear (V_S_) wave velocities are affected within fluid-saturated media (supplementary information). We implemented a numerical scheme for evolution of backfill material by building relationships among the seismic and physical properties of the backfill material and saturating fluids. Through which the temporal changes in Vp, Vs and Vp/Vs ratio in a backfill material can be precisely accounted.

**Fig 1:**
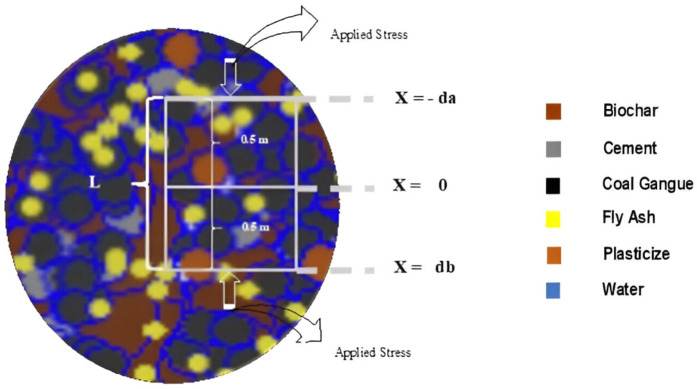
Pictorial representation of considered model and the representative element for numerical simulation.

In the current study, we considered a single fluid in the form of water enclosed in a porous rock. This chamber contains water-saturated rock, which is subjected to a force in the form of stress exerted by the strata above when a wave pass through subsurface media. We considered a represented element (RE) in the form of a rectangle having “L” length in total. The upper end of the RE is placed in such a way it enclosed an area (a) whose thickness is 0.5 m, and the lower end is placed in such a way it enclosed an area (b) with a thickness of 0.5 m (Fig 1). To maintain the undrained condition within the RE, a condition of no flow of fluid from any boundary of the RE is made sure. It was also considered that the stress, pore pressure, fluid and solid velocity is continuous between the two layers (a, b). Due to the movement of fluid caused by applied stress “$\tau_{⊥}$ ”, the effective stress “$P_{e}$ ” will displace both fluid and solid which will cause strain “ε ” within the rock sample.

### Poroelastic relationships

Divorkin [28] established the relationship of stress and fluid pressure with the solid and fluid displacement, given below;


$$\begin{array}{c} \tau_{⊥}=\;(P^{\prime }+Q^{\prime })u_{s}+(Q^{\prime }+R^{\prime })U_{f}\\ p_{f}=-(Q^{\prime }\;u_{s}+R^{\prime }U_{f})/\phi \end{array}$$ (1)

In above equations,


P′=Kd+4μ3+(α−ϕ)2Ka,Q′=ϕ(α−ϕ)KaR′=ϕ2KaKa=α−ϕKs+ϕ/ϕKf\nulldelimiterspaceKf,α=1−KdKs (2)

Whereas, $K_{d}\;,\;\mu \;,\;\phi \;$ stands for the dry bulk modulus, the shear modulus and the porosity of the surrounding rock, $K_{s}$ indicates the mineral’s modulus $K_{f}$ is demonstrating the fluid’s bulk modulus. The suggested system was viewed as a wholly enclosed system in which there wasn’t any flow via any representing element’s border (top/bottom, right/left). A wave flowing through the representational element will apply the stress ${\hat{p}}_{e}$, which will cause the representational element to become strained ε. The mathematical relationship seen between yield stress and the resulting strain, where there is complete stress-strain consistency, can produce the P-wave modulus ($H^{\prime }$), and It has the following definition:


$$H^{\prime }=\frac{-{\hat{P}}_{e}}{\varepsilon }$$ (3)

Whereas ${\hat{p}}_{e}$ is the applied stress exerted from the above strata, and ∊ is the resultant strain. Furthermore, the computed modulus ($H^{\prime }$) is connected with the effective P and S-wave numbers as:


kp_eff=ω*ρe/ρeH′\nulldelimiterspaceH′ks_eff=ρe/ρeμ\nulldelimiterspaceμ (4)

In above equations ‘μ ’ represents the shear modulus of rock medium, the existence of pores and fissures will significantly lower the rock moduli. The Biot-consistent theory [29], which describes this effect, can be used to compute its magnitude. The real part of P and S wave number will give the compressional (V_P_) and shear wave (V_S_) velocity respectively, and then we will ultimately compute V_P_/V_S_.


$$\begin{array}{c} V_{p}=\omega /Re\mathrm{\backslash nolimits}\_k_{p\_eff}\\ V_{s}=\omega /Re\mathrm{\backslash nolimits}\_k_{s\_eff} \end{array}$$ (5)

Where $\rho_{e}$ is a representation of the effective medium density [30–32], and it can be characterized as:


$$\rho_{e}=\sum_{m=a,b}\frac{L_{m}}{L}\rho_{m}$$ (6)

Where, $\rho_{m}\;=\;(1-\phi_{m})\rho_{sm}+\phi_{m}\rho_{fm}$ is the density of the respective layer m, $\rho_{sm}$ and $\rho_{fm}$ are indeed the densities of the solid and fluid phases in a porous medium “m ” bearing the porosity $\phi_{m}$.

Our aim is to quantify the variation in Vp/Vs ratio at different composition, for that the Biot poroelastic theory offers an appropriate framework for fluid-saturated media [26,27]. The poroelastic equation of motion is given by Dutta and Od´e [33]:


$$\begin{array}{c} \rho_{b}\frac{\partial^{2}u_{s}}{\partial x^{2}}+\;\;\rho_{f}\frac{\partial^{2}W}{\partial t^{2}}=\;(P^{\prime }+2Q^{\prime }+R^{\prime })\frac{\partial^{2}u_{s}}{\partial x^{2}}+\frac{Q^{\prime }+R^{\prime }}{\phi }\frac{\partial^{2}W}{\partial x^{2}}\\ \rho_{f}\frac{\partial^{2}u_{s}}{\partial x^{2}}+\frac{\rho_{b}}{\phi^{2}}\frac{\partial^{2}W}{\partial t^{2}}=\;\;\;\frac{Q^{\prime }+R^{\prime }}{\phi }\frac{\partial^{2}u_{s}}{\partial x^{2}}\;\;+\frac{R^{\prime }}{\phi^{2}}\frac{\partial^{2}W}{\partial x^{2}}-\frac{b}{\phi^{2}}\frac{\partial W}{\partial t} \end{array}$$ (7)

where ϕ is the porosity of subsurface media, $\rho_{f}$ is the fluid density, $\rho_{s}$ is the solid density, $\rho_{b}=\left[(1-\phi )\rho_{s}+\phi \rho_{f}\right]$ is fluid-solid coupled density, $u_{s}$ is the displacement in solid, W is the fluid-solid coupled motion $W=\phi (u_{s}-U_{f})$, $U_{f}$ is the displacement in fluid, and P′, Q′, and R′ are constants to vary with the dry bulk modulus $K_{d}$, shear modulus (μ), porosity (ϕ) of surrounding rock, mineral bulk modulus $K_{s}$, and fluid bulk modulus $K_{f}$. The solution of the Biot’s equations can be expressed in the following way Dutta1979:


$$u=u(x)e^{iwt},\;W=W(x)e^{iwt}$$ (8)

Additionally,


$$W=W_{c}+W_{d},\;u=u_{c}+u_{d}\;$$ (9)

In the previously given relationships, ‘$u_{c}=\sigma_{d}W_{c}$ ’ and ‘$u_{d}=\sigma_{c}W_{d}$ ’ and one can write the theory of Biot’s formulation in its decoupled form calculations [33] stated in the following form:


$$(\frac{\partial^{2}}{\partial x^{2}}{+k}_{c}^{2})W_{c}(x)=0\;\;,\;\;(\frac{\partial^{2}}{\partial x^{2}}{+k}_{d}^{2})W_{d}(x)=0\;$$ (10)

where, the wave numbers $k_{c}$, $k_{d}$ can be given as follows:


kc2/kc2w2\nulldelimiterspacew2=(σ11c4−σ21c2+iμc2/κw)/(c1c4−c2c3)kd2/kd2w2\nulldelimiterspacew2=(σ12c3−σ22c1+iμc1/κw)/(c2c3−c1c4) (11)

The following are the parameters related to wave number:


$$\begin{array}{c} \sigma_{11}=\rho_{b}\sigma_{c}+\rho_{f}\\ \sigma_{12}=\rho_{b}\sigma_{d}+\rho_{f}\\ \;\sigma_{21}=\rho_{f}\sigma_{c}+m\;\;\\ \;\sigma_{22}=\rho_{f}\sigma_{d}+m\;\;\\ c_{1}=\;(P^{\prime }+2Q^{\prime }+R^{\prime })\sigma_{c}+(Q^{\prime }+R^{\prime })/\phi \\ c_{2}=\;(P^{\prime }+2Q^{\prime }+R^{\prime })\sigma_{d}+\;(Q^{\prime }+R^{\prime })/\phi \\ c_{3}=(Q^{\prime }+R^{\prime })\sigma_{c}/\varphi \;+\;R^{\prime }/\phi^{2}\\ c_{4}=(Q^{\prime }+R^{\prime })\sigma_{d}/\varphi \;+R^{\prime }/\phi \\ W_{c}=K1cos(k_{c}x)+K2\sin(k_{c}x)\\ W_{d}=K3cos(k_{d}x)+K4\sin(k_{d}x) \end{array}$$ (12)

In above equations, $k_{c}$, $k_{d}$ represents the corresponding wave numbers for compression and dilatation. Also, the coupled fluid-solid and solid displacement can be express as follows:


$$W=K1cos(k_{c}x)+K2\sin(k_{c}x)+K3cos(k_{d}x)+K4\sin(k_{d}x)$$ (13)

$$u=\sigma_{c}K1cos(k_{c}x)+\sigma_{c}K2\sin(k_{c}x)+\sigma_{d}K3cos(k_{d}x)+\sigma_{d}K4\sin(k_{d}x)$$ (14)

Whereas, K1 - K4 are variable to be known, applying adequate boundary conditions will allow us to calculate these variables. In these boundary conditions, the stress, fluid pressure, coupled fluid-solid and solid displacement should indeed exist in continuous form between layers a, and b.

### Boundary conditions

To solve the above equations, the first boundary condition was that, the applied stress and pore pressure along with solid and fluid-solid coupled velocity was considered to be continuous at the boundary between layer (a) and (b) i.e. at x = 0


atx=0

$$\tau_{⊥\;a}=\tau_{⊥\;b}$$ (15)

$$\;\;a\;=\;p_{\;b}$$ (16)

$$\;\;\phi \;(u_{a}-u_{a})\;=\;\;\phi \;(u_{b}-u_{b})$$ (17)

$$u_{a}=\;u_{b}\;$$ (18)

Furthermore, in order to maintain the undrained boundary condition, the applied stress, and the pore fluid pressure is considered to be continuous, also the fluid-solid coupled velocity is zero at the top and bottom end of the RE.


$$at\;x=-d_{a}$$

$$\;\;\;\tau_{⊥\;a}=-p$$ (19)

$$\begin{array}{c} \phi \;(v_{a}-u_{a})\;=\;\;0\;\\ at\;x=d_{b} \end{array}$$ (20)

$$\;\;\tau_{⊥\;b}=-p$$ (21)

$$\phi \;(v_{b}-u_{b})\;=\;\;0$$ (22)

The aim of implementing the boundary conditions was to obtain the equations that have a link between stress, pore fluid pressure, fluid-solid coupled velocity, and the velocity of the solid. These equations will additionally contain the unidentified parameters (K1 − K4). Therefore, once we have these unidentified parameters, we may arrange them into matrix as shown under:


$$\sum_{i,j=1}^{8}A_{i,j}B_{i}\;\;=\;\;C_{i}$$ (23)

Where, A_ij_ is a matrix having coefficients mentioned in equation (13 − 14) which can be obtained by implementing above boundary conditions to equations 1, and 13–14, B is a column vector having following unidentified parameters:


B=(K1,K2,K3,K4,K5,K6,K7,K8)T (24)

Whereas, C is a vector indicating the tension placed on the edges of the rectangular box, given in the form as follows:


$$\;C\;\;=\;\;p_{e}(\;-1,0,0,0,0,0,-1,0\;{)}^{T}\;$$ (25)

After adding the solutions for B and C in equation (23), the matrix will be solved, and help to measure the solid displacements $u_{a}$ and $u_{b}$ which will ultimately help to calculate the total strain ∊ within the representative medium. Afterwards, the necessary P-wave modulus (H′), could be acquired. Through the computed modulus the V_P_ will be computed whereas the V_S_ will be computed through the shear modulus as given in equation 4. Finally, the V_P_/V_S_ ratio will be computed.

To get the input parameters, i.e., modulus of elasticity for dry rock ($K_{d}$, $\mu_{d}$) in terms of porosity, for both numerical and analytical calculations, the methodology adopted by [29] is employed.


Kd=Kr(1−ϕ/ϕϕc\nulldelimiterspaceϕc),μd=μr(1−ϕ/ϕϕc\nulldelimiterspaceϕc) (26)

In above equation, $K_{f}$ is the bulk modulus of the fluid = 2.2GPa, $K_{d}$ is the bulk modulus and $\mu_{d}$ is the shear modulus of dry rock with empty pores, ϕ is the porosity of the rock, $\phi_{c}$ is the critical porosity that is set as 0.18, $K_{r}$ is the bulk modulus and $\mu_{r}$ is the shear modulus of the rock matrix.

### Model and input parameters

The model’s accuracy will be ensured by estimating the elastic moduli (Bulk and Shear modulus) of the backfill materials Voigt-Reuss-Hill averaging technique. i.e., The Voight (upper bound and Ruess (lower bound) [34]. We considered a backfill material comprises of a mixture of fly ash, coal gangue, biochar, cement, plasticizer and water (Table 1).

Voight upper bound


$$\begin{array}{c} K_{v}=\sum_{i=1}^{n}f_{i}K_{i}\\ G_{v}=\sum_{i=1}^{n}f_{i}G_{i} \end{array}$$ (27)

Ruess lower bound


$$\begin{array}{c} \frac{\text{1}}{K_{R}}\;=\;\sum_{i=1}^{n}\frac{f_{i}}{K_{i}}\\ \frac{\text{1}}{G_{R}}\;=\;\sum_{i=1}^{n}\frac{f_{i}}{G_{i}} \end{array}$$ (28)

Through the above mathematical relations, we will measure the elastic moduli of the backfill material, which will further help us to measure the Vp and Vs velocities through such materials which will lead us to measure the Vp/Vs ratios of the material and ultimately the effectiveness of the backfill material will be identified. The simulation involves analyzing the responses of the backfill materials under various stress and saturation conditions by numerically solving Biot’s equations. The proposed approach examines the induced stress, strain, and displacement within the heterogeneous medium, shedding light on the mechanical interactions and potential failure mechanisms. Furthermore, by utilizing the above equations, we compute the dynamic behavior of backfill material over time, by considering different chemical compositions and variable stress conditions. The help us to achieve our aim to evaluate material’s stability and integrity throughout the drying process. Also the aim to predict the evolution of pore geometry with the passage of time, we further analyze the variations in Vp, Vs and Vp/Vs by applying the locally weighted scatter plot smoothing (LOWESS) method in Matlab and ensured a smooth representation of velocity trends as drying progresses.

## Results and discussion

Since our approach is based on numerical simulation, we considered a homogeneously mixed material at fined scale and ensured the uniform response in its seismic properties. Through implementation of above numerical methodology, we calculated the compressional wave velocity by taking into account the time-dependent change in the parameters of backfill materials (Table 2).

**Table 2:** Summary of parameters as a function of material composition.

Water		
K_f_ = 2.2 GPa Water bulk modulus	ρ_f_ = 1000 kg m^-3^ Water density	η_f_ = 6*10−4 kgm^-1^s^-1^ Viscosity of water
**Composite of Fly ash, cement, gangue, biochar**		
K_s_ = ~18.6 GPa	ρ_s _= ~1700–2000 kg m^-3^	µ = ~0.2–12 GPa
K_d _= ~7.89 GPa	ɸ = 0.07–0.13	κ = 1*10^-11^m^2^

**Fig 2:**
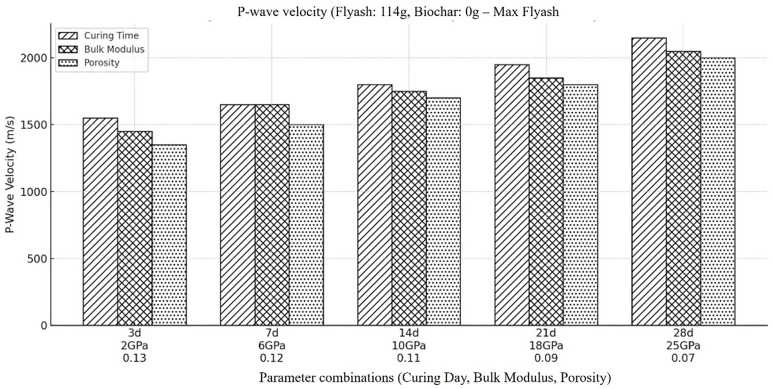
Compressional wave velocity within the backfill material, when there is higher amount of fly ash in it (i.e., Fly ash (114g), Coal gangue (150g), Biochar (0g), Cement(30g), Plasticizer(6g), Water (87g).

Specifically, we looked at a saturated bulk modulus range of 1–25 GPa and a changing porosity range from 0.07 to 0.13 as the materials age. To precisely estimate the material’s mechanical behavior over time. We further take into account the changing characteristics of fly ash and biochar as well as their interactions throughout the curing process. by adjusting the percentages of these materials while maintaining the other component constant, we determined materials overall behavior with the passage of time. The results are presented below:

Compressional wave velocity (Vp) within the backfill material is numerically computed, when there is max concentration of flyash (114g) and lowest concentration of biochar (0g) in it (Table 2). The above figure (Fig 2) represents a texture-coded bar-charts demonstrating the change in P-wave velocity due to variation in porosity and bulk modulus over time (3–28 days) during curing process of backfill material. An increase in P-wave velocity trend over time can be clearly observed in the outcomes. The possible reason of this behavior could be; backfill material usually becomes denser due to porosity reduction and elastic properties get improved as material cures with time, especially if it contains cement or other similar binding agents. An extensive analysis of the changes in porosity over time reveals that this is predominantly due to access of flyash as it contributes to stiffness and strength of material over time and due to drying of cement, which results in a denser and less porous structure (Mohapatra & Rao, 2001). The process of drying can cause changes in the compressional wave velocity (Vp) of backfill materials, particularly those containing cement and having binding ability. Hydration, a chemical reaction between water and cement particles that forms a hardened structure, is the process by which cement is cured. From the figure it can be concluded that the mechanical strength of backfill material’s get enhance over time. The increased in bulk modulus, decrease in porosity and rise in P-wave velocity clearly indicate curing dynamics and material suitability for use in backfill mining process.

**Fig 3:**
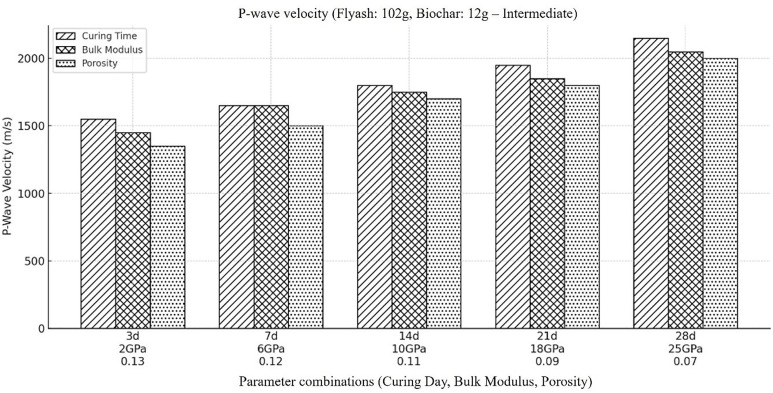
Compressional wave velocity within the backfill material, when there is moderate amount of fly ash in it (i.e., Fly ash (102g), Coal gangue (150g), Biochar (12g), Cement(30g), Plasticizer(6g), Water (87g).

Next, the influence of decrease in fly ash (from highest 114g to intermediate 102g) with an increase in biochar concentration (from lowest 0g to intermediate 12g) on the P-wave velocity is computed (Fig 3). As the material cures, a similar pattern of increasing P-wave velocity is observed in the outcomes, as we saw for composites with more fly ash and less biochar content. However, there is a decrease in the overall compressional wave velocity (Vp) as illustrated in Fig 3. The observed behavior can be explained by the fact that a lower fly ash concentration would lead to less pozzolanic reaction and, as a result, a stiffer and less solidified material (Eshun et al., 2018). In comparison to the situation with the maximum fly ash content, a lower quantity will result in increased porosity, decrease in the Vp can frequently be the outcome of this increase in porosity, since there will be additional openings containing water or air, which are less efficient at propagating compressional waves.

In addition, biochar behaves less rigid than the fly ash along with having higher porosity values, this ultimately results in higher overall porosity of the backfill material with the increase in the amount of biochar. Furthermore, biochar has more structural varied makeup and potential weak spots due to its heterogeneous composition which ultimately results in decreased Vp. The properties of biochar being less stiff and having less binding strength then the fly ash make give rise in attenuation of wave and ultimately results in decreased compressional velocity. So, adding more biochar means creating more heterogeneity and allowing compressional waves to get dispersed, because less homogeneous material give rise to disrupted wave, lower Vp will be the outcome.

**Fig 4:**
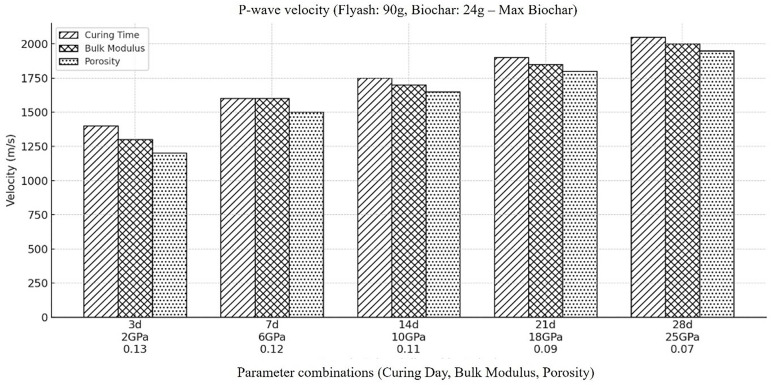
Represents the velocity of compressional wave when the amount of fly ash decreased to its lowest concentration in the backfill material (i.e., Fly ash (90g), Coal gangue (150g), Biochar (24g), Cement(30g), Plasticizer(6g), Water (87g).

To further explore the variation in material characteristics with time, a maximum concertation of biochar along with lowest fly ash concentration is considered and the behavior of compressional wave velocity (Vp) within such backfill material is calculated (Fig 4). From the outcomes, a noticeable decrease in overall velocity of compressional wave within a backfill material whose concentration for fly ash is get decreased and concentration for biochar get increased. The decrease in Vp can be attributed to the lesser amount of pozzolanic compound formation owing to lower fly ash concentration, which eventually leads to increased porosity and decreased stiffness. Addition of biochar, which is known for being porous and lacking of mechanical rigidity is the other factor which added its contribution to overall decrease in Vp. As mentioned above the increase in biochar concentration results in increase in heterogeneity of the material and the resultant heterogeneous structure restricts the efficient transmission of compressional wave velocity, which is further intensify by the dispersion and attenuation due to the presence of biochar particles. The material’s limited capability for mechanical strengthening is maintained throughout time by the biochar’s continual porosity and lack of responses to pozzolanic material. According to the results, the cement hydrates and greatly increases the mixture’s density and stiffness, which causes the sample’s compressional wave velocities to rise as it dries. We can conclude that, in order to completely understand the influence on velocities of compressional wave (Vp) due to variation in concentration of fly ash and biochar while keeping other component constant, we must consider material’s properties and their interaction over time. The conclusions emphasize the importance of proper optimization of backfill material composition during mining operations, so that the stability and mechanical properties required for backfill material can be ensured.

**Fig 5:**
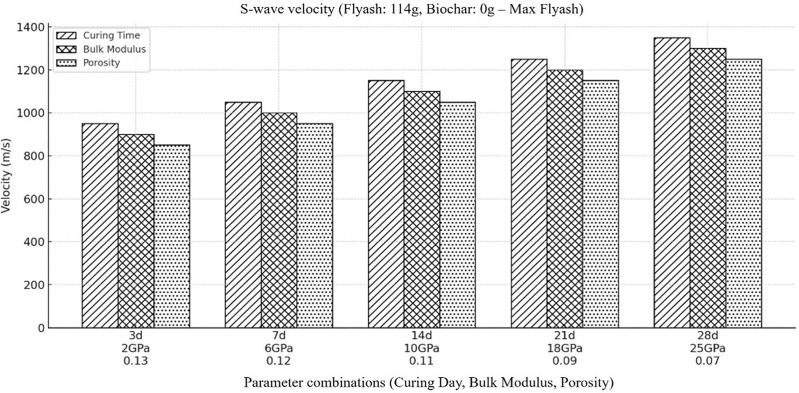
Represents the velocity of shear wave within the backfill material, when the amount of fly ash is at its highest concentration (i.e., Fly ash (114g), Coal gangue (150g), Biochar (0g), Cement(30g), Plasticizer(6g), Water (87g).

In order to comprehensively investigate the proper optimization of backfill material composition for mining operations, high concentration of fly ash with zero concentration of biochar is considered and conducted a detailed analysis to compute the shear wave velocity (Vs) within the backfill material. The outcomes (Fig 5) reveal that Vs increases gradually over time at high fly ash and zero biochar concentration. The significant decrease in porosity with time as the sample get dry explain this trend, the decrease in porosity results in decrease in proportion of air or water filled pores which ultimately give rise in shear wave velocity. In addition, the densification and consolidation of mixture get increased due to higher fly ash concentration, which results in higher Vs values. Increase in densification and consolidation results in higher stiffness (T. Chen et al., 2024; Zhao et al., 2018) which allows shear wave to propagate through the material more efficiently and ultimately results in increased shear wave values. These research findings emphasizes that fly ash plays a significant role in enhancing the mechanical properties and overall integrity of backfill materials used in mining operations. This will eventually result in substantially larger and more robust solutions for backfilling.

**Fig 6:**
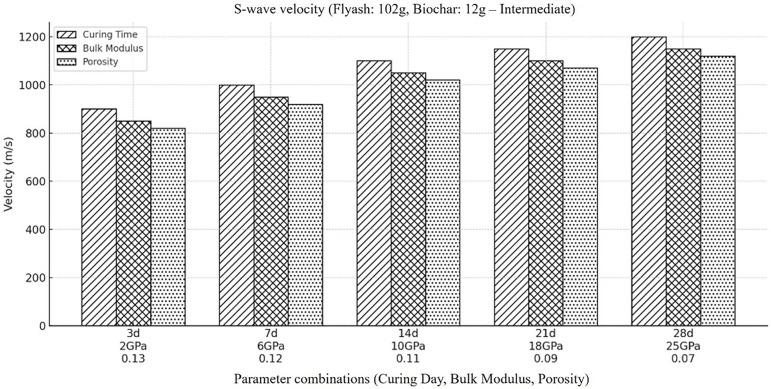
Represents the velocity of shear wave within the backfill material, when the amount of fly ash is at its moderate concentration (i.e., Fly ash (102g), Coal gangue (150g), Biochar (12g), Cement(30g), Plasticizer(6g), Water (87g).

In order to further Investigate the influence of the variation in backfill material on the shear wave velocity (Vs) a detailed analysis of the material under varying concentrations of fly ash and biochar is required. So, the shear wave velocity was then calculated on the assumptions that the concentration of fly ash was going to decrease from its highest value to an intermediate value and the concentration of biochar was to rise from its zero concentration to an intermediate concentration. The outcomes displayed in Fig 6, indicate that the overall shear wave velocity get decrease with the decrease in fly ash and increase in biochar concentration, the inclusion of extra amount of biochar, give rise to decline in stiffness and density of backfill material which ultimately cause the decrease in shear wave velocity. The growing heterogeneity in the backfill material produce by inclusion of biochar cause significant scattering and attenuation of shear wave which ultimately results in decrease in shear wave velocity. Furthermore, the outcomes reveal that Vs tends to increase over time and are in line with observations of high fly ash and low biochar concentration. As describe before the time dependent increase in Vs may be due to growing stiffness and consolidation of the material, which are more evident in the presence of high fly ash content. Additionally, shear wave propagation gets improved and significantly contribute to the overall mechanical integrity and stiffness of the mixture when the pozzolanic reactions in fly ash mixture get enhanced.

**Fig 7:**
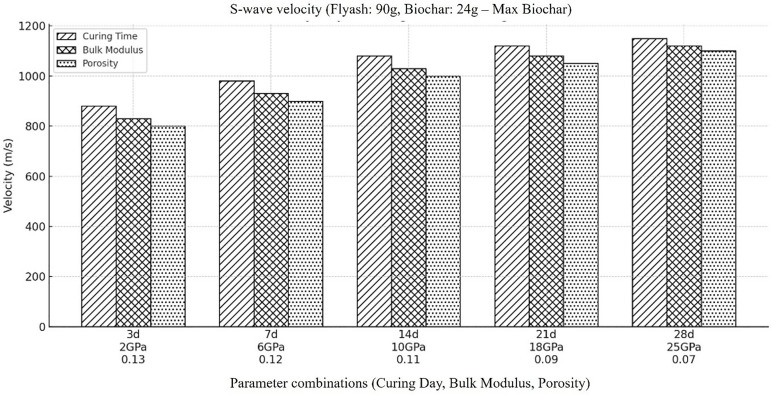
Represents the velocity of shear wave velocity when the amount of fly ash decreased to its lowest concentration in the backfill material (i.e., Fly ash (90g), Coal gangue (150g), Biochar (24g), Cement(30g), Plasticizer(6g), Water (87g).

Next, we examined a backfill material that had the highest concentration of biochar and the lowest percentage of fly ash, taking into account the calculation of shear wave velocity (Vs) across various time intervals. The findings show that (Fig 7) with the decrease in concentration of fly ash and increase in concentration of biochar the velocity of shear wave get decrease consistently. This demonstrates that the overall Vs decreases as fly ash concentrations increase and biochar concentrations decrease. Additionally, the time dependency of the increase in shear wave velocity is consistent across all concentrations of fly ash and biochar. As describe before for previous cases, many factors could be involved in the apparent decrease in overall shear wave velocity of the material as the concentration of biochar get increase and the concentration of fly ash get decrease. One of the main reasons could be the highly porous and heterogenous nature of biochar which cause a decrease in overall density and stiffness of the material which ultimately resist in propagation of shear wave. The high porosity results in increase in dispersion and attenuation of shear wave and ultimately cause decrease in shear wave velocity.

However, the time dependent increase in shear wave velocity throughout all the combination of biochar could be the result of decreased porosity owing to slow consolidation and compaction of the backfill material. Additionally, as the material get dry with time the interparticle contact improves which ultimately enhance the overall mechanical integrity and stiffness of the material. The role of residual fly ash with any concentration within the material cannot be ignored as it may still undergo pozzolanic reaction strengthens the stiffness of the material. From the outcomes of the shear wave velocity results, we can conclude that to precisely account the dynamics of shear wave velocity due to varying fly ash and biochar concentration, a careful consideration of fly ash and biochar ratio should be considered to get the best mechanical properties for certain mining operations.

**Fig 8:**
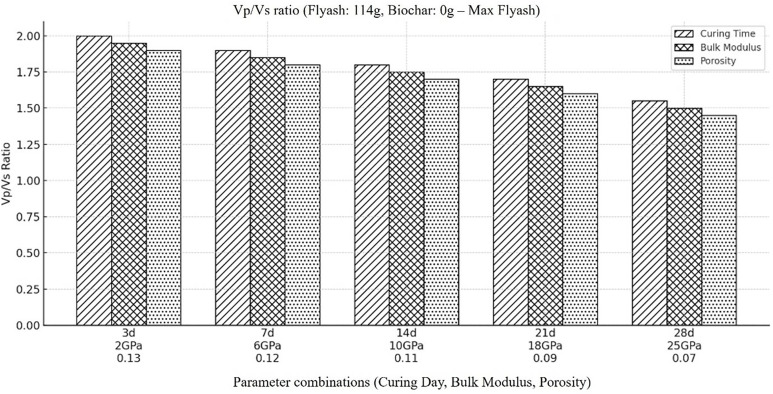
Represents the ratio of compressional to shear wave velocity (Vp/Vs) within the backfill material, when the amount of fly ash is at its highest concentration (i.e., Fly ash (114g), Coal gangue (150g), Biochar (0g), Cement(30g), Plasticizer(6g), Water (87g).

Based on the chemical compositions of the backfill material shown in Table 1, the simulated backfill material’s compressional (Vp) and shear (Vs) wave velocities considering medium’s changing porosity as a result of temporal variations in its properties. From the results it is evident that both the compressional and shear wave velocities results in higher values when highest concentration of fly ash and lowest concentration of biochar is considered. The Vp/Vs ratio for the material with the greatest fly ash concentration and the lowest biochar content was also determined, taking these temporal variations into account. The results, as indicated in Fig 8, it can be concluded that due to the substantial reduction of porosity and increase in stiffness caused by fly ash, the findings show a lower Vp/Vs ratio for this composition. While both compressional and shear waves propagate more quickly as a result of reducing porosity and increasing stiffness. Shear wave velocity is often more significantly impacted by the stiffness increase, indicating the increase in strength of the material with the passage of time due to low fluid saturation and compaction with time. Consequently, increased mechanical durability and reduced permeability are the results of fly ash’s primary effect on stiffness and porosity reduction. Because of its increased stiffness and decreased porosity, the material with the greatest fly ash and lowest biochar concentration is therefore more suited for backfilling applications, offering lower permeability and higher mechanical qualities.

**Fig 9:**
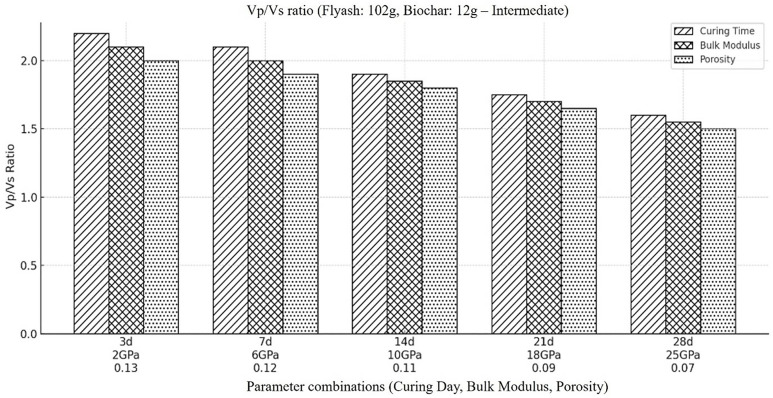
Represents the ratio of compressional to shear wave velocity (Vp/Vs) within the backfill material, when the amount of fly ash is at its intermediate concentration (i.e., Fly ash (102g), Coal gangue (150g), Biochar (12g), Cement(30g), Plasticizer(6g), Water (87g).

Following that, we calculated the Vp/Vs ratio for the backfill material that contained a moderate quantity of biochar and fly ash. Fig 9 demonstrates that when the total amount of fly ash is decreased to an intermediate level, while the concentration of biochar is increased to an intermediate level from the original values, the overall Vp/Vs ratio rises. There could be several reasons for this rise in the Vp/Vs ratio. First reason could be the ability of fly ash to both enhance and reduce the porosity of the material, so decreasing fly ash concentration means decreasing the overall stiffness of the material ultimately decreasing the shear wave velocity which is more sensitive to the stiffness of the material then the compressional wave velocity, so overall Vp/Vs ratio will get increase. In the meantime, addition of more porous and flexible material biochar means reducing the stiffness and porosity results in increasing the Vp/Vs ratio. Increase in amount of fluid with the addition of biochar could be another reason for the increase in Vp/Vs ratio, because the ability of biochar to hold more fluid due its high porosity gets increase with the addition of biochar and reduction of fly ash. In combination, these modification in material reduce compressional wave velocity but the decrease in shear wave velocity is more rapid and ultimately give rise in (Vp/Vs) ratio of the material. Such composition reflect a material that could be more porous and fluid saturated so less strong in term of its mechanical behavior then the one with higher fly ash concentration due to its less porosity and high mechanical strength.

**Fig 10:**
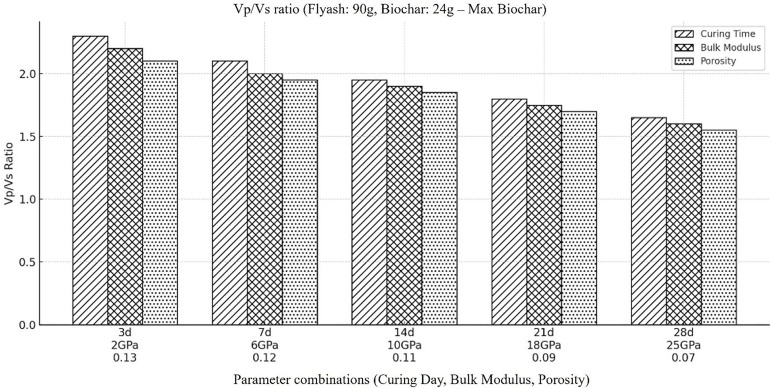
Represents the ratio of compressional to shear wave velocity (Vp/Vs) within the backfill material, when the amount of fly ash is at its least concentration in it (i.e., Fly ash (90g), Coal gangue (150g), Biochar (24g), Cement(30g), Plasticizer(6g), Water (87g).

In order to further strengthen our interpretation of previous results, we further computed the ratio of compressional to shear wave velocity (Vp/Vs) by considering the combination of highest biochar and lowest fly ash concentration in backfill material. The outcomes (Fig 10) reveals that the combination of decrease in fly ash concentration and increase in biochar concentration results in increased Vp/Vs ratio of the material. These outcomes validate our earlier conclusion for the combination of intermediate concentration of fly ash and biochar and allow us to deeply understand how variation in these elements combinedly influence the propagation characteristics of compressional and shear wave.

Many factors can be considered as the reason of increase in Vp/Vs ratio with the increase in biochar concentration and decrease in fly ash concentration. For example, flyash have the ability to reduce the porosity and increase the stiffness of the material so decreasing fly ash concentration means increasing porosity and decreasing stiffness. As shear wave velocity is more sensitive to stiffness of the material, so decreasing flyash concentration will result in decreasing stiffness and ultimately in decreasing shear wave velocity and increase in Vp/Vs ratio.

Conversely, biochar is more porous than the flyash, so increasing biochar means increasing overall porosity of the material providing more space to accumulate the fluids in it and its property of being low dense results in decrease in stiffness of the material which ultimately effect Vs and have less effect on Vp, so the overall ratio of compressional to shear wave velocity Vp/Vs will increase.

Based on above observations, the increased in Vp/Vs ratio in a material with low fly ash and high biochar concentration can thus be attributed to the simultaneous effect of decreased stiffness and increased porosity. Additionally, the holding capacity of fluid content will increase with the increase in biochar and decrease in fly ash concentration which will lead to increase in compressional velocity while the shear velocity continuously decreases due to reduced stiffness of the material with presence of fluids.

From the above interpretation, we get to know that speedy propagation of shear wave significantly depends on the amount of fly ash, as it largely contributes in strengthening the backfill material, on the other hand, biochar is more capable of fluid accumulation which leads to a comparatively higher reduction in shear wave velocity. The decrease in Vp/Vs ratio with time at different fly ash and biochar concentration is consistent in any combination indicating the increased stiffness of the material with time due to reduction in porosity and decrease in amount of fluid. Drawing a conclusion from the above discussion we came to know that the backfill material composition required to be carefully adjusted so that the proper stability of material can be achieved for its proper use.

## Conclusions

For the overall assessment and analysis of backfill materials that may be utilized in various civil engineering and mining fields, recent research has been conducted to ascertain and examine the impact of different concentrations of fly ash and biochar. These investigations are particularly concerned with their effect on elastic wave velocities, both compressional wave velocities (Vp) and shear wave velocities (Vs), and Vp/Vs ratios of the backfill material in question. It was found through an investigation that with high percentages of fly ash in a material and low percentages of biochar, this combination yields much higher stiffness, a significant decrease in porosity, and an improvement in overall mechanical behavior. This is well reflected through the observation of higher values of Vp and Vs and a reduced Vp/Vs ratio. Conversely, it was found that with higher percentages of biochar, this results in porosity increase while at the same time reducing the shear wave velocity. These alterations in material properties eventually increase the Vp/Vs ratio and compromise the material’s mechanical strength. The intermediate concentrations provide a reasonable compromise between various possibilities but are less effective compared to the mixtures with high fly ash concentrations. In summary, comparing mechanical stability and structural strength in backfill mixtures, it is clear that the mix with high fly ash content combined with low biochar content is the most suitable.

In order to maximize and expand the scope of this research, the viability of using the Vp, Vs and Vp/Vs ratio as an in-situ monitoring tool should also be explored since it is capable of offering real-time measurements of the mechanical integrity and development of backfill throughout its lifespan. This would enable the facile and flexible implementation of the research outputs under various geological conditions and mines.
